# Mechanistic Relevance of Ventricular Arrhythmias in Heart Failure with Preserved Ejection Fraction

**DOI:** 10.3390/ijms252413423

**Published:** 2024-12-14

**Authors:** Pegah Bahrami, Kelly A. Aromolaran, Ademuyiwa S. Aromolaran

**Affiliations:** 1 Nora Eccles Harrison Cardiovascular Research and Training Institute (CVRTI), University of Utah School of Medicine, 95 S 2000 E, Salt Lake City, UT 84112, USA; u6061560@utah.edu (P.B.); kelly.aromolaran@utah.edu (K.A.A.); 2 Department of Surgery, Division of Cardiothoracic Surgery, Nutrition & Integrative Physiology, Biochemistry & Molecular Medicine Program, University of Utah School of Medicine, Salt Lake City, UT 84112, USA

**Keywords:** obesity, diabetes, heart failure, sudden cardiac death, ventricular, arrhythmias, ion channel remodeling

## Abstract

Heart failure with preserved ejection fraction (HFpEF) is increasing at an alarming rate worldwide, with limited effective therapeutic interventions in patients. Sudden cardiac death (SCD) and ventricular arrhythmias present substantial risks for the prognosis of these patients. Obesity is a risk factor for HFpEF and life-threatening arrhythmias. Obesity and its associated metabolic dysregulation, leading to metabolic syndrome, are an epidemic that poses a significant public health problem. More than one-third of the world population is overweight or obese, leading to an enhanced risk of incidence and mortality due to cardiovascular disease (CVD). Obesity predisposes patients to atrial fibrillation and ventricular and supraventricular arrhythmias—conditions that are caused by dysfunction in the electrical activity of the heart. To date, current therapeutic options for the cardiomyopathy of obesity are limited, suggesting that there is considerable room for the development of therapeutic interventions with novel mechanisms of action that will help normalize sinus rhythms in obese patients. Emerging candidates for modulation by obesity are cardiac ion channels and Ca-handling proteins. However, the underlying molecular mechanisms of the impact of obesity on these channels and Ca-handling proteins remain incompletely understood. Obesity is marked by the accumulation of adipose tissue, which is associated with a variety of adverse adaptations, including dyslipidemia (or abnormal systemic levels of free fatty acids), increased secretion of proinflammatory cytokines, fibrosis, hyperglycemia, and insulin resistance, which cause electrical remodeling and, thus, predispose patients to arrhythmias. Furthermore, adipose tissue is also associated with the accumulation of subcutaneous and visceral fat, which is marked by distinct signaling mechanisms. Thus, there may also be functional differences in the effects of the regional distribution of fat deposits on ion channel/Ca-handling protein expression. Evaluating alterations in their functional expression in obesity will lead to progress in the knowledge of the mechanisms responsible for obesity-related arrhythmias. These advances are likely to reveal new targets for pharmacological modulation. Understanding how obesity and related mechanisms lead to cardiac electrical remodeling is likely to have a significant medical and economic impact. Nevertheless, substantial knowledge gaps remain regarding HFpEF treatment, requiring further investigations to identify potential therapeutic targets. The objective of this study is to review cardiac ion channel/Ca-handling protein remodeling in the predisposition to metabolic HFpEF and arrhythmias. This review further highlights interleukin-6 (IL-6) as a potential target, cardiac bridging integrator 1 (cBIN1) as a promising gene therapy agent, and leukotriene B4 (LTB4) as an underappreciated pathway in future HFpEF management.

## 1. Introduction

Heart failure (HF) with preserved ejection fraction (HFpEF), defined as a state of diastolic dysfunction accompanied by objective evidence of functional or structural cardiac abnormalities and a left ventricular ejection fraction (LVEF) exceeding 50%, is a complex clinical condition and is the most prominent form of HF among the elderly [1]. Despite the substantial increase in HFpEF at a rate of 1% a year, there is a critical paucity of rational therapeutic interventions, suggesting that the increasing burden of HF/HFpEF will likely continue to increase globally [2,3]. This lack of progress may be because HFpEF is heterogeneous in nature, comprising different phenotypes mainly derived from its various comorbidities, leading to HFpEF presenting with a wide range of clinical presentations [4]. Existing preclinical animal models that focus on only a single phenotype may not fully capture the complexity of the cellular remodeling processes involved in HFpEF. Therefore, there is an unmet clinical need to improve our current understanding of the underlying mechanisms of HF.

There is increasing evidence in the extant literature that HF/HFpEF mortality in patients occurs in 53% to 74% of all HFpEF cases in the first 5 years after diagnosis [2]. Randomized clinical trials of HFpEF patients have shown an increased risk of sudden cardiac death (SCD) in patients [5]. However, the underlying mechanisms involved are unknown. The major drivers of SCD are known to be lethal cardiac arrhythmias. While ventricular arrhythmias (VAs) are the most common cause of SCD in HF with reduced ejection fraction (HFrEF), it remains to be determined whether it is indeed VA or the progression of HF itself that causes SCD in the HFpEF population [6,7]. Reports from implantable loop recorders in patients with HFpEF and HF with mid-range ejection fraction (EF: 40–49%) have demonstrated a high incidence of non-sustained ventricular tachycardia (NSVT), but not sustained VT, in this population [8]. Similarly, clinical studies using ambulatory electrocardiogram (ECG) monitoring and pacemaker recordings observed a notable prevalence of NSVT in HFpEF patients [9,10]. While the incidence of NSVT seems to be a common feature in HFpEF in these studies, the presence of an association between NSVT with morbidity, mortality, and SCD differs between studies [8,9]. Nevertheless, ventricular tachyarrhythmias encompass a significant percentage of in-hospital deaths and cardiac arrests in HFpEF patients [7,11]; therefore, their influence on HFpEF-associated SCD demands further attention.

The putative mechanisms governing the development of VA in HFpEF include deteriorated conduction velocity and reentry circuits stemming from hypertrophic and fibrotic ventricles, delayed repolarization, and altered excitation–contraction coupling [12]. Major cardiac currents play pivotal roles in maintaining the integrity of the electrical activity of the heart. This electrical activity can be defined by the spatiotemporal patterns of the action potential phenotypes as well as cardiac refractoriness [13]. In that regard, preclinical studies have shown that the downregulation of potassium (K) currents and delayed repolarization have important roles in the increased susceptibility to VA and, subsequently, SCD in rats fed with a high-salt diet who developed HFpEF [14,15].

**Figure 1:**
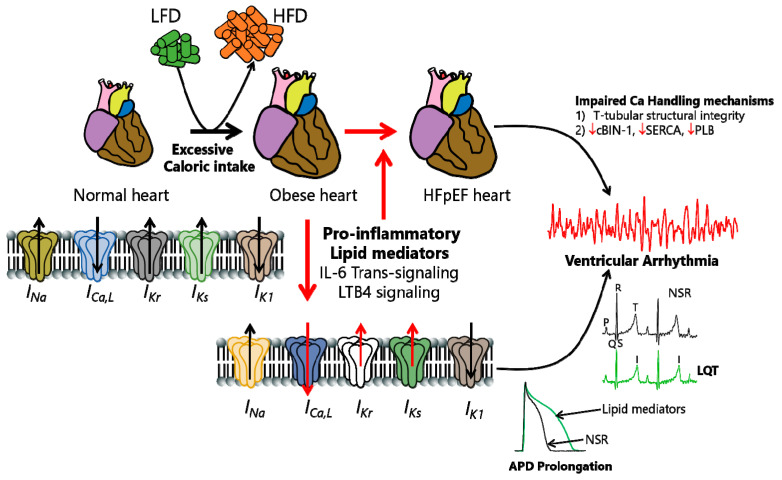
Cartoon illustration showing hypothesized obesity-linked channelopathies leading to pathological APD prolongation, LQT prolongation, ventricular arrhythmias, and ultimately, increased risk of sudden cardiac death in obese patients with HFpEF. We speculate that overactivated obesity-linked heightened proinflammation—possibly through individual or multiple combinations of IL-6 trans-signaling or LTB4 pathways—contributes prominently to the pathogenesis of HFpEF. The exact cellular proarrhythmic effects of IL-6 and LTB4 involved are poorly understood. We suspect that overactivated IL-6- and LTB4-signaling-linked remodeling processes may occur through altered gene and protein expressions of ion channel subunits and biophysical defects (subunit trafficking and/or gating defects). Whether and how *I_Na_* and *I_K_*_1_ are modulated in obesity-linked IL-6 and LTB4 signaling or HFpEF is unknown. We further speculate that understanding the contributions of distinct cellular arrhythmia triggers that are sensitive to obesity/lipotoxic mechanisms is important for interpreting the mechanistic bases of how obesity channelopathies promote life-threatening VA/SCD risk and inform targeted anti-arrhythmia therapy in patients. HFpEF may promote structural changes in the organizational integrity of Ca-handling proteins (including cBin-1, SERCA, and PLB) and contribute to VA/SCD risk. Interestingly, there is a growing interest in the potential beneficial cardiovascular impact of cBIN1 gene therapy in preserving the functional and structural integrity of Ca-handling mechanisms in HF, with significant implications for HFpEF. The modulation of ventricular ion channels by cBIN-1 is currently unknown, suggesting that there is significant room for improving the current therapeutic interventions in obese HF/HFpEF patients.

Targeting both the underlying pathways and comorbidities is essential for further advancements in HFpEF arrhythmia management. Obesity is considered a major comorbidity of HFpEF and is characterized as a metabolic condition with chronic low-grade inflammation [16]. Growing evidence suggests that obesity and associated pathologies (metabolic syndrome and insulin resistance) are also important contributors to the development of HFpEF [17]. Obesity is further associated with a prolonged corrected QT interval (QTc), a marker of ventricular relaxation, and is also found to be a contributing factor in HFpEF-associated VA [11,18]. In that regard, pathological changes in ionic currents or channelopathies may prominently contribute to an increased risk of VA/SCD in obese/HFpEF patients. Therefore, it would be interesting to discern the impact of a functional interplay between obesity triggers, as a prominent HFpEF comorbidity, and pathological ion channel functional expression (Figure 1) as well as how they affect disease outcomes in patients. In this regard, an improved understanding of the cellular proarrhythmic mechanisms of remodeled ion channels may be a key component for the rational development of safe and effective interventions in obese patients with HF and, particularly, HFpEF. Therefore, this review aims to provide an overview of the factors linking HFpEF, obesity, and ventricular arrhythmias by exploring the underlying mechanisms of the disturbances in electrical currents and channelopathies (Table 1). Lastly, we further summarize the significant and unappreciated pathways underlying functional and structural changes in HFpEF, which can potentially pave the way for future advances regarding VA treatment in this disease.

For the purpose of this review, search engines that included the PubMed Central and Google Scholar databases were used to search for studies published in the English language. Our searches were not limited by date restrictions, were free texts, and included the following keywords: “heart failure”, “heart failure with preserved ejection fraction”, “diastolic dysfunction”, “sudden cardiac death”, “obesity”, “lipid mediators”, “leukotrienes”, “ventricular arrhythmias”, “dyslipidemia”, “cardiac calcium channel”, “cardiac potassium channel”, “cardiac sodium channel”, “Ca handling proteins”, “SERCA2a”, “RyR2”, “phospholamban”, “pro-inflammatory cytokines”, “interleukin signaling”, “leukotrienes B4 signaling”, and “cBIN1”.

## 2. Interconnections Between Obesity, HFpEF, and VA

Targeting obesity as a therapeutic approach in HFpEF management has recently gained significant attention due to the success of the STEP-HFpEF [34]/STEP-HFpEF DM [35] and SUMMIT [36] trials in HFpEF patients with obesity receiving semaglutide and tirzepatide, respectively. Semaglutide and tirzepatide are anti-diabetic and weight-loss medications that belong to the classes of glucagon-like peptide-1 (GLP-1) and dual glucose-dependent insulinotropic polypeptide (GIP)/GLP-1 receptor agonists, respectively [34,36]. In addition to improved physical function and quality of life, long-term administration of these medications also lowered the mortality caused by cardiovascular events and HF progression [34,35,36]. This breakthrough in HFpEF management further highlights the significance of obesity and metabolic conditions in HFpEF.

Obesity moderates diastolic dysfunction by triggering the core pathophysiological factors of HFpEF, such as oxidative stress and inflammation [37,38]. Excessive fat accumulation and the secretion of certain adipokines are among the underlying mechanisms through which NADPH oxidases (NOXs) are produced in obesity [39]. NOX is a fundamental source of hydrogen peroxide (H_2_O_2_), a major reactive oxygen species (ROS) behind obesity-induced oxidative stress [39]. Moreover, elevated NOX activity results in enhanced endothelial nitric oxide synthesis (eNOS) uncoupling, which in turn produces increased levels of superoxide, another major ROS, instead of nitric oxide (NO). Superoxide rapidly binds to NO, forming peroxynitrite, further exacerbating ROS production and reducing NO bioavailability. Furthermore, obesity and metabolic syndrome bring about a state of metabolic inflammation in which damaged hypertrophic, hyperplastic, and hypoxic adipocytes provoke the accentuated production and release of inflammatory cytokines like interleukins 1, 6, and 23 (IL-1, IL-6, and IL-23); tumor necrosis factor-alpha (TNF-α); transforming growth factor-β (TGF-β) [37,40]; and adipokines and promote macrophage polarization toward the M1 proinflammatory phenotype [37,38]. It should be noted that although increased levels of cytokines may be initially cardioprotective, they become detrimental as inflammation persists [37].

Similarly, elevated expressions of H_2_O_2_ and NOX are observed in HFpEF [41]. The microvascular structure in HFpEF exhibits coronary endothelial inflammation and signs of oxidative stress, providing constant stimulation through elevated inflammatory cytokines [37]. Eventually, this systemic microvascular inflammation causes endothelial dysfunction, which was well demonstrated in an investigation on obese HFpEF rats and also HFpEF patients that revealed the presence of eNOS uncoupling and a decline in NO bioavailability in endothelial cells and macrophages under oxidative stress [41].

Obesity has been related to VA development through heightened levels of free fatty acids (FFAs), inducing dysregulation in ventricular repolarization and resulting in a prolonged Q-T interval [42]. Guinea pig models fed with a high-fat diet (HFD) serve as an inflammatory preclinical model and have shown increased susceptibility to VA [33]. HFD-fed preclinical models have been extensively used to study the effects of obesity on ion channels and currents. Dysregulations in Na, Ca, and K currents in the context of obesity and high FFAs have been linked to altered cardiac excitability, long QT intervals, and VA, particularly in response to an enhanced sympathetic trigger, in mice and guinea pigs [33,43,44]. Moreover, epicardial adipose tissue (EAT) has recently been identified as a possible promoter of VA in addition to its already established involvement in atrial arrhythmias [45]. Although advances have been made in identifying obesity-related VA-inducing factors, much remains to be discovered regarding the precise mechanism of how major cardiac ion channels are modulated in metabolically disrupted HFpEF. A thorough comprehension of the pathways binding obesity with HFpEF and VA is crucial for filling the gap in these patients, and this will have clinical and pharmaceutical implications.

## 3. Pathophysiology and Promotion of Molecular Changes in Cardiac Ionic Currents by VA

Ion channel dysfunction remains one of the crucial factors in VT initiation. The normal ventricular cardiac action potential (AP) is defined as follows: Phase 0, due to a large inward sodium current (*I_Na_*) is followed by Phase 1, which is shaped by rapid activation and inactivation of transient outward K^+^ currents (*I_to_*) [46]. Subsequently, ionic currents due to voltage-gated L-type calcium (Ca) (*I*_*Ca*,*L*_) and the Na–Ca exchanger (*I_NCX_*) channels and the slow component of delayed rectifier current (*I_Ks_*) create the plateau phase of the AP [47]. In addition, a small inward sodium current called the late *I_Na_* contributes to the plateau phase [48]. Repolarization is controlled by the slow (*I_Ks_*) and rapid (*I_Kr_*) components of the delayed rectifier current (*I_K_*). The resting membrane potential is controlled by the inward rectifier *K* current (*I_K_*_1_) [49]. Thus, decreases in outward currents [50,51] or increases in depolarizing mechanisms [52] delay repolarization, resulting in the prolongation of the heart-rate-corrected QT interval (QT_c_), which predisposes patients to fatal VT, such as torsades de pointes (TdP) [53] and SCD [54,55].

Understanding the dynamics of cardiac ionic currents and channels in HFpEF holds significant importance because they can influence therapeutic approaches and drug development. For instance, *I_Kr_* remains the dominant channel of clinical cardiotoxicity; the FDA and European Medicines Agency require screening against the human ether-a-go-go-related gene (hERG) for all new drugs being evaluated [56,57]. Therefore, decreases in *I_Kr_* prolong the ventricular action potential duration (APD) [51,58,59,60] and predispose patients to an elevated risk for early afterdepolarizations (EADs) [61,62], a prolonged QT_c_ interval, and TdP [63]. In addition, impaired function of *I_Ks_*, *I_Na_* (peak and late), and *I_Ca_* currents contribute to arrhythmia risk [64,65,66,67], highlighting the importance of multi-ion-channel analyses, which may inform the rational development of safer approaches (with reduced cardiotoxic effects) to anti-arrhythmic monotherapy and polytherapy for patients.

Cardiac AP occurs due to finely tuned interactions between intracellular structures and ionic currents that stimulate the excitation and contraction of ventricular cardiomyocytes [68]. A prolonged AP is a prominent phenomenon observed in HFpEF and ventricular arrhythmias [14]. Assessment of AP using patch-clamp electrophysiology [69] and optical mapping with voltage-sensitive dyes [70,71] has become an important tool in studying cardiac electrophysiology and arrhythmogenicity in preclinical animal models. In the clinical setting, action potential phases are demonstrated as an ECG [72]. The QRS complex and Q-T interval constitute ventricular depolarization and repolarization, respectively, and their variations serve as indicators of the imminent onset of ventricular arrhythmias [72]. The ventricular tissue’s triggered activity manifests as EADs and delayed afterdepolarizations (DADs) [72]. While an increase in *I*_*Ca*,*L*_ current is thought to be the main contributor in both, they differ in timing, as EADs occur in phase 2 (prolonged AP duration) or 3 (abbreviated AP duration), and DADs take place in the resting stage of the action potential [63,73]. Furthermore, afterdepolarizations and abnormalities in calcium handling may promote the formation of reentry circuits, which are arrhythmogenic conditions where an AP repeatedly moves in a closed-circle manner [74]. Dispersion of action potentials throughout the heart is defined as varying repolarization times in different regions of the heart and is believed to be able to cause reentry circuits and eventually VA [75]. Despite the significant role of ionic changes in triggering VA, a definite therapeutic option has yet to be proposed that prompts a deeper investigation into the molecular basis of ionic changes.

### 3.1. Voltage-Gated Sodium Channels and Sodium Currents

Voltage-gated Na channels play a pivotal role in cardiac excitation and are key modulators of the AP. Na channels consist of one pore-forming α-subunit and non-pore-forming auxiliary beta-subunits [76]. Cardiac Na channels are composed of various isoforms, with the sodium channel protein type 5 subunit alpha (Nav1.5) being the most abundant [77]. The Nav1.5 channel is encoded by the SCN5A gene and is highly expressed in the conduction tissues of the atria, ventricles, and Purkinje fibers [78]. Mutations or autoimmune dysregulations of the Nav1.5 channel are the putative mechanisms behind serious arrhythmic syndromes like long QT syndrome and Brugada syndrome [79,80], which may instigate lethal VAs and TdP and lead to SCD [81]. Nav1.5 channels are responsible for the rapid inward sodium currents entering the cell at phase 0 of the AP [78]. While this rapid influx of Na and its subsequent upstroke in AP constitute the largest component of Na currents, a late *I_Na_* current, although smaller in magnitude, greatly influences the duration and morphology of the AP due to the sustained depolarization it creates in phases 2 and 3 [82,83]. Late Na currents occur due to the late physiological inactivation of a number of Nav1.5 channels, and their density is increased in HFpEF [19]. Although dysregulations in both peak and late Na currents contribute to cardiac arrhythmias [84], an increase or “gain of function” in late *I_Na_* is accountable for the development of cardiac diastolic dysfunction by reversing the function of the NCX, allowing Ca to enter the cell, inducing a decline in myocyte relaxation and triggering DADs [85,86]. Subsequently, these alterations lead to a delay in repolarization and a prolonged AP and Q-T interval, creating an arrhythmogenic environment [87], which has been demonstrated to underlie the incidence of lethal VA in rat models of HFpEF [12,14].

The pivotal role of late *I_Na_* in HFpEF and associated dysrhythmias is further confirmed by the ability of late *I_Na_*-inhibiting drugs such as ranolazine [88,89,90] to decrease NCX expression [85] and enhance diastolic function in HFpEF patients [91]. However, it should be noted that, although echocardiographic changes in these patients were significantly in favor of enhanced diastolic function, conflicting evidence exists regarding the effect of ranolazine in reversing the elongated QT interval in HFpEF [91]. More recently, sodium–glucose cotransporter-2 (SGLT2) inhibitors such as empagliflozin and dapagliflozin have emerged as promising treatment options in HFpEF patients, exhibiting lower overall hospitalization and CVD rates [92,93]. Moreover, empagliflozin has demonstrated protective clinical effects against VA in diabetic patients [94,95]. To delve deeper, preclinical animal and in vitro investigations have been conducted to evaluate their roles in late *I_Na_* and VA [19,96], demonstrating a reversal of late *I_Na_* in long QT syndrome mutations of Nav1.5 with empagliflozin, dapagliflozin, and canagliflozin [96] as well as proarrhythmic AP changes in HFpEF models, particularly with empagliflozin [19]. Notably, these three SGLT2 inhibitor drugs display selective inhibition of late *I_Na_*, meaning that they display few to no effects on peak *I_Na_* [96]. Philippaert et al. also confirmed the ability of empagliflozin to diminish late *I_Na_* current in an HF mouse model that had undergone transverse aortic constriction (TAC) [96]. Their study, however, does not indicate whether the animals had reduced or preserved EF [96]. In addition, it is known that although the pressure overload caused by TAC can result in hypertrophy, it is not a promising model for HFpEF [97]. Adding subcutaneous deoxycorticosterone acetate (DOCA), a mineralocorticoid agent, just after TAC surgery in mice can lead to a successful mimic of HFpEF at both the structural and physiological levels [98]. Considering the significance of the selective inhibition of late *I_Na_* in HFpEF outcomes, it can be proposed that future studies should assess the effects of dapagliflozin and canagliflozin in HFpEF-mimicking animal models such as DOCA+TAC mice [98] or HFD-fed guinea pigs [33]. Nevertheless, much remains unknown regarding proarrhythmic changes in HFpEF, and the identification of the underlying molecular mechanisms regulating late *I_Na_* currents to better understand the risk factors precipitating resistance or sensitivity to medications is crucial.

Phosphorylation of Nav1.5 channels by kinases is a crucial modulator of late *I_Na_* physiology [87]. The calcium–calmodulin kinase (CaMK) II family and its cardiac predominant isoform, CaMKIIδc, are of paramount significance in this phosphorylation, given their overexpression in human and animal HF models [99]. In vivo, overexpression of CaMKII has been linked to shortened effective refractory periods, prolonged repolarization duration, and increased inclination for ventricular tachycardia [100]. Furthermore, the application of the autocamtide-2-related inhibitory peptide (AIP, a CaMKII inhibitor) reportedly reverses AP prolongation by inhibiting CaMKII in obese HFpEF mice [19], supporting the arrhythmogenic behavior of CaMKII. Notably, the same experiment demonstrated that myocytes with CaMKII proteins carrying oxidative-resistant mutations (MM281/282VV) are less influenced by H_2_O_2_-induced late *I_Na_* [19].

There is an intricate relationship among intracellular Na and Ca levels, reactive oxygen species (ROS), and CaMKII, in which an elevated intracellular Na^+^ level leads to ROS production in the mitochondria, which eventually activates and stimulates CaMKII and ryanodine receptors (RyRs), thereby repeating the cycle [101]. Thus, oxidative stress and an inflammatory state may precipitate VA through intracellular Na, Ca, and late *I_Na_* modulation [101], and it may be the reason behind the prolonged late *I_Na_* observed in obese mouse models of HFpEF in the study by Hegyi et al. [19]. Still, no alterations were observed in Na currents in rats with metabolic syndrome [20], contradicting this hypothesis. Nevertheless, unlike other ions and currents, less is known about the mechanisms underlying how these cytokines influence Na concentration and currents.

### 3.2. Voltage-Gated L-Type Calcium Channels, Calcium Currents, and Calcium-Handling Proteins

The coordination between voltage-gated L-type calcium channels and calcium-handling proteins is crucial for the proper functioning of the excitation–contraction (EC) coupling machinery [102]. The alpha-conducting subunit of Cav1.2, which is the dominant form of voltage-gated L-type calcium channel in cardiac muscle, is encoded by the CACNAC1 gene [103]. RyRs (or calcium-release channels) and sarcoplasmic reticulum Ca-ATPase (SERCA) play a vital role in regulating intracellular calcium concentrations (Table 2) [104]. Briefly, the I_*Ca*,*L*_-current-mediated increase in calcium concentrations initiates the opening of the RyR2 channel, leading to the release of calcium from the sarcoplasmic reticulum (SR), a phenomenon known as Ca-induced Ca release (CICR) [105]. This calcium release activates Ca-sensing troponin C, initiating contraction. Relaxation occurs as cytosolic calcium levels decrease, facilitated by both the SERCA-mediated Ca reuptake into the SR and the NCX-mediated Ca extrusion into the extracellular space [106]. Dysregulation in Ca–EC coupling is one of the main pillars of VA pathophysiology in HFpEF patients [12]. The alterations in EC and intracellular Ca concentrations are typically characterized by either an increased release of Ca into the sarcoplasm through Cav1.2, transient receptor potential (TRP), RyR2, or inositol 1,4,5-trisphosphate receptor type 2 (IP3R2) or the impaired uptake of Ca by NCX or SERCA2a [107]. Although the mechanisms behind EC changes are well established in HFrEF, the parts that play more important roles in HFpEF are not well understood.

In contrast to HFrEF, Ca transient amplitudes are generally heightened in HFpEF [23,31]. While an overexpression of Cav1.2 proteins was identified in DAHL salt-sensitive HFpEF rat models by Kilfoil et al. [23], Ca recruitment by RyR2 is deemed to be a more prominent and observed moderator of Ca influx into the cytosol in other HFpEF models [23,31,111]. RyR2-mediated Ca leakage into the cytosol instigates diastolic Ca waves known as Ca sparks [31]. This enhanced extraction of Ca from the SR by RyR2 channels may be due to the increased or maintained density of transverse tubules (T-tubules) in HFpEF [110], contrasting with HFrEF [112]. T-tubules are invaginations of the surface membrane that are predominantly found in ventricular myocytes and are abundant in proteins involved in EC and Ca handling [113]. A higher density of T-tubules has been linked to higher intracellular Ca concentrations [113] and underlies the adaptive mechanism of HFpEF toward a stiff myocardium [110,112]. An elevated density of T-tubules is negatively correlated with the distance to the sarcoplasmic reticulum, contributing to faster Ca release, which may explain the reduced latency attributed to Ca release exhibited in the high-salt-fed rat models of HFpEF in a study by Kilfoil et al. [23,110]. Additionally, RyR2 phosphorylation by either protein kinase A (PKA) or CaMKII may also be an underlying feature leading to the aberrant release of Ca into the cytosol; however, debate persists as to which of these kinases are responsible for this phenomenon and to what extent [112,114]. Nevertheless, dantrolene, a muscle relaxant that has shown preventive behavior toward CPVT in a CPVT mutant knock-in mouse model [115], has been identified as facilitating the affinity of calmodulin, a calcium-sensitive protein that inhibits RyR2 activity, to RyR2 in mild hypertensive HFpEF rats, hence attenuating Ca release [111].

Cellular calcium transient kinetic phenotypes (including amplitude and decay) are another fundamental mediator of pathological Ca responses and associated arrhythmogenesis. Phospholamban (PLB) serves as a critical and reversible modulator of SERCA that inhibits SERCA2a in its dephosphorylated state, thereby reducing its affinity for Ca ions and curbing Ca decay by the SR [116]. This mechanism is, however, reversed by PKA- or CaMKII-mediated phosphorylation of PLB [117]. Disruptions in the PLB–SERCA2a relationship are portrayed as a dysregulated SERCA2a–PLB ratio in favor of SERCA2a inhibition, leading to perturbed calcium uptake by the SR and accumulated cytosolic Ca [118]. Agreeing with this notion, the hypertensive HFpEF rats presented by Rouhana et al. exhibited a prolonged Ca decay time and an increased PLB-to-SERCA2a ratio, which led to enhanced diastolic Ca levels; in addition, enhanced contractility performance was demonstrated by longer sarcomere length (SL) shortening times and lower *I_K_*_1_ currents in phase 4 [31]. Nonetheless, the electrophysiological mechanism of Ca-handling systems is complex, and no one HFpEF model can fill all gaps. In particular, SERCA2a promotion may also lead to a long QRS interval and VA as a result of the release of Ca into the cytosol [25]. Interestingly, in a normotensive hypertrophic rat model with diastolic dysfunction, while both SERCA2a and PLB proteins were found to be expressed at a low level, both were phosphorylated at the CaMKII site, indicating SERCA2a activation and PLB inhibition [25]. This augmented Ca decay, therefore, prompted heightened Ca sensitivity and release—probably through RyR2 channels [25].

The contributions of obesity and metabolic syndrome to Ca current alterations, which elevate susceptibility to VA, are more prominent than their contributions to Na current alterations. Obesity exerts Ca-handling remodeling through various pathways and mechanisms, mainly encompassing PLB [26,27,30,108] and mitochondrial Ca cycling [108,119] alterations. Obesity not only hinders PKA-mediated PLB phosphorylation [26,109] but is also associated with elevated PLB mRNA expression in obese Wistar rats [30]. However, contrasting data exist regarding PLB protein expression [28]. The influence of obesity on Ca-handling proteins and the L-type calcium channel is seemingly more pronounced at the mRNA level rather than the post-translational levels [22,26,27,28,30]. Mitochondrial Ca cycling in HFpEF is distinct from that in HFrEF, as mitochondrial Ca concentrations are increased, unlike the decreased values in systolic dysfunction [108]. In the obese diabetic and hypertensive rat models in a study by Miranda-Silva et al., Ca mitochondrial levels as well as cytosolic concentrations were elevated both during contraction and at rest [108]. Therefore, it can be concluded that a close relationship exists between mitochondrial and cytosolic Ca concentrations in HFpEF. High mitochondrial Ca levels likely compensate for the high contractility performance through ATP production, as indicated by the slow sarcomere shortening [108]. However, persistently elevated levels of Ca in the mitochondria open the apoptotic-inducing mitochondrial permeability transition pores (mPTPs), which, in turn, can stimulate the release of Ca from the SR into the cytosol. This state of Ca overload will eventually trigger the formation of Ca waves, where each Ca efflux into the cytosol triggers a rapid Ca reuptake by SERCA and then a greater Ca efflux by RyR2 [120,121]. Ca waves can potentially lead to DADs and cardiac arrhythmias [120]. Gordan et al. discovered that mice with diminished mPTP function exhibited a lower incidence of Ca waves and were protected against cardiac arrhythmias [122]. Moreover, there is evidence that a high-fat diet induces mPTP opening [123]. Hence, the inhibition of these pores may present a viable solution for VA in metabolism-associated HFpEF.

### 3.3. Voltage-Gated Potassium Channels and Potassium Currents

Several distinct classes of K channels are present in the ventricular tissue, and they are responsible for the flow of associated K currents and shaping the AP in different phases. Voltage-gated K channels, which are responsible for rapid repolarization and transient outward K currents (*I_to_*), are encoded by KCND2/3 and KCNA4 [124,125]. The hERG, KCNQ1, and KCNE1 genes encode α-subunits of voltage-gated K channels controlling the final repolarization of the membrane potential through rapid (*I_Kr_*) and slow (*I_Ks_*) K currents (KCNQ1 and KCNE1) [125,126,127]. Lastly, the inward rectifier K currents (*I_K_*_1_), responsible for setting the resting membrane potentials, are encoded by the KCNJ12/14/4 genes [125,128].

A decline in K currents is reportedly linked to HFpEF and associated VA. An in vitro investigation in high-salt-fed HFpEF rat cardiomyocytes revealed delayed repolarization and reduced *I_to_*, *I_Kr_*, and *I_K_*_1_, leading to prolonged QTc and APD, which was confirmed with ex vivo optical mapping [14]. Moreover, HFpEF rat models were more inclined to develop VT, ventricular fibrillation (VF), premature ventricular contractions (PVCs), and SCD, which is believed to be the result of reentry circuits [14]. Reentry circuits are the result of delayed repolarization due to decreased K currents [14]. Delayed repolarization creates an environment of varying refractoriness in the heart, allowing for opportunistic functional reentrant signals [14]. Similarly, a reduction in *I_K_*_1_ and *I_to_* was observed in obese diabetic mice who received subcutaneous aldosterone infusions and eventually demonstrated HFpEF [21]. Isolated ventricular cardiomyocytes of these animals were further treated with empagliflozin, AIP, and vericiguat (an FDA-approved medication for severe HFrEF that acts by promoting the soluble guanylate cyclase pathway [129]), leading to reduced AP prolongation and APD alternans among all HFpEF mice exposed to empagliflozin and AIP, as well as in female mice treated with vericiguat [21]. Given the promising results in alleviating these arrhythmogenic changes, future studies can be proposed to investigate their effects on K currents and channels.

Disruptions in voltage-gated K channels predispose the heart to arrhythmias and increase the likelihood of abnormal electrical patterns [130]. Mutations in KCNQ1 and the subsequent dysregulation in slow delayed rectifier channels are closely linked with long QT syndrome [131,132]. The activation and deactivation of slow delayed rectifier K currents are slow at rest; however, once stimulated, their activation becomes faster, while deactivation remains slow [133]. This allows their associated mutations and downregulations to be closely linked to lethal VA, such as TdP [133]. However, this phenomenon is limited to sympathetic stimulation; therefore, medications targeting *I_Ks_* have not yet yielded any success [134].

While all ventricular K currents exhibit decreased transients in preclinical models of HFpEF [14,31], the most prominent ion channel activity change observed in HFpEF is the functional downregulation of the *I_to_* current, which is critical to determining early repolarization [14,135,136]. However, exogenous activation of *I_to_* currents, although beneficial in reversing QTc prolongation, has failed to attenuate arrhythmias in HFpEF, which may be due to the downregulation of the cardiac channel responsible for *I_to_* (Kv4.3) found in cardiomyocytes [14]. The simultaneous reduction in KCND2/3 and Kv4.3 highlights the significance of investigating K channel protein-encoding genes at both the transcriptional and protein expression levels [14].

There is a paucity of studies on how obesity regulates pathological K current functional expression and ultimately continues to VA in HFpEF. However, there is evidence that obesity regulates K currents through lipotoxicity and cytokine release [127]. Palmitic acid (PA) is a saturated fatty acid and a promoter of lipotoxicity that induces the secretion of proinflammatory cytokines, including IL-1β, IL-6, and TNFα [137]. Assessments of ventricular myocytes from guinea pigs fed with high-fat diets that included PA showed prolonged AP and QT intervals, likely through individual or multiple combinations of proinflammatory cytokines, including IL-18, IL-1β, and IL-6, as well as TNFα-induced cardiac remodeling [33,137]. As reduced expression of KCNQ1, KCNE1, and ERG-1a (*I_Kr_* channel encoded by hERG) was detected in these myocytes, it was evident that lipotoxicity and obesity led to alterations in the potassium channels, allowing for lower *I_Kr_* and *I_Ks_* densities [33,137]. Notably, preincubation of non-obese guinea pig ventricular myocytes with IL-6 and IL-6/IL-6 receptors (IL-6Rs) showed a similar decline in ERG expression, with the latter having a more prominent decrease [58]. Corbin et al. further elucidated that the inhibition of *I_Ks_* in obese guinea pigs resulted in a higher risk of a more severe VA once exposed to isoproterenol [33]. However, investigations by Haim et al. on ventricular myocytes from palmitate-fed mice yielded contrasting results, showing elevation in all outward K currents [32]. This contrast likely lies in the major differences in major repolarizing K currents between mice and humans, prompting the search for ideal preclinical cardiometabolic models that can accurately mimic human conditions [138].

Beyond voltage-gated K currents, other cardiac K currents may play a role in the development of HFpEF. Intriguingly, inhibition of TREK-1 in fibroblasts of TWIK-related potassium channels (TREK-1), members of the cardiac two-pore domain K channels (K2P), in fibroblasts, exhibited a protective effect against pressure overload [139,140,141]. Further research is needed to assess the role of TREK-1 in VA, as fibrosis is believed to induce reentry circuits by disrupting normal electrical conduction [12,142].

## 4. Uncovering Potential Therapeutic Targets

Despite the promising results exhibited by anti-diabetic and weight loss medications in overall HFpEF prognosis, their precise anti-arrhythmic mechanisms and effects on ion channel modulations are still not fully discovered. Therefore, uncovering the underlying cellular mechanisms and identifying new therapeutic targets for HFpEF is still an urgent need. The complex pathophysiology of HFpEF is the fundamental reason underlying the failure to reach suitable therapeutic options and is why the medications proven beneficial in HFrEF fail to provide significant outcomes in HFpEF [143,144,145]. Cardiometabolic disturbances and associated comorbidities drive HFpEF by disrupting the endothelial structure. Indeed, HFpEF is not a single disease summarized by a preserved systolic function. Thus, the therapeutic approach to HFpEF also needs to cover both the structural and functional aspects of the disease [146]. Among the various aspects shaping the pathophysiology of HFpEF, inflammation and Ca-handling disturbances in cardiomyocytes are of great importance. Among inflammatory cytokines, IL-6 is of paramount importance given its ability to reduce *I_Kr_* and thereby modulate ventricular repolarization [58]. Further, dapagliflozin has demonstrated significant efficacy in reducing IL-6 levels [147], and empagliflozin administration in diabetic HFrEF patients has also resulted in a marked reduction in circulating levels of this cytokine [148]. On the other hand, cardiac bridging integrator 1 (cBIN1) has been discovered as a crucial structural protein responsible for T-tubule organization and Ca handling in the SR [149]. cBIN1 is decreased in HF, and improvements in both systolic and diastolic function are observed in cBIN1 gene transfer into the pressure-overload HF mice [150], rendering cBIN1 a hopeful candidate for gene therapy investigations in HFpEF. Therefore, understanding the cellular mechanisms of IL-6 and cBIN1 as potential therapeutic targets of inflammatory pathways and structural disturbances in HFpEF is crucial. In addition, leukotriene B4 (LTB4), a bioactive lipid, is a strong inflammatory agent involved in excessive FFA production and lipotoxicity promotion [151] that is found to be able to decrease *I_Kr_* [152]. However, lipid mediators and LTB4 in particular are an understudied facet in HFpEF and cardiac arrhythmias that require further attention.

### 4.1. Interleukin-6 Signaling in Heart Failure

IL-6 is a pleiotropic cytokine (downstream of IL-1β action) [153] that serves as the core of cardiometabolic cytokine signaling [154], and it is a powerful predictor of the severity of heart diseases [155,156,157]. IL-6 has been associated with the development of atherosclerosis [158], angiotensin-2-mediated formation of unstable atherosclerotic plaques and unstable angina [159,160], increased myocardial infarct size 4 months post-ST elevated myocardial infarction (STEMI) [161], and elevated incidence of major adverse events and mortality in patients with coronary diseases [162]. Although less clinical evidence exists regarding the role of IL-6 in HFpEF, recent studies have demonstrated that IL-6 seems to be playing a crucial role in the prognosis of HFpEF and is positively associated with higher readmission rates and mortality [163,164]. Moreover, observations suggest that a marked elevation of IL-6 holds an intricate link to excess body fat, body mass index (BMI), and obesity in patients with HFpEF [165,166,167], further agreeing with the proinflammatory consequences of lipotoxicity [168].

Classical IL-6 signaling occurs through its membrane-bound receptor (IL-6Rα)–glycoprotein 130 (gp130) receptor complex, and it mediates homeostasis and regenerative functions [156,169]. The soluble IL-6R (sIL-6R) is generated through extracellular shedding or alternative processing of the mRNA encoding IL-6R [170]. The proinflammatory effects of IL-6 are mediated via trans-signaling, whereby IL-6 binds to sIL-6R [171,172] and engages gp130 in target cells [173], leading to the activation of the downstream JAK/STAT and SHP2/ERK signaling pathways [174,175]. Prolonged obesity and HFD intake are well associated with elevated IL-6 trans-signaling [176]. Given that higher IL-6 concentrations in HFpEF patients are correlated with augmented levels of other inflammatory markers like TNFα and high-sensitivity C-reactive protein (hs-CRP) [177], it can be concluded that IL-6 trans-signaling may play a major role in IL-6 signaling pathways in HFpEF, as opposed to classical signaling. However, the potential therapeutic benefits and efficacy of selectively targeting IL-6 trans-signaling for the prevention of HF are unknown. There is, therefore, a critical need to define the cellular mechanisms of IL-6 and determine the therapeutic potential of selective anti-IL-6 trans-signaling in preclinical models of HF. Without such information, the promise of a novel class of anti-IL-6 therapies for the treatment of human HF will likely remain unfulfilled.

Notably, in a study by Hagiwara et al., cardiomyocytes derived from SHP2-dysregulated gp130 mutant mice that had been pre-incubated in an IL-6- and IL-6R-containing medium did not show increased Ca current density in a whole-cell patch-clamp analysis in comparison with wild-type mice in the same environment [175]. In vitro studies have elucidated that IL-6 may play a role in cardiac diastolic dysfunction through the downregulation of SERCA2 [178,179,180] and can lead to increased *I_Ca_*, L-type current density, and Ca transients [175]. Treatment of HEK-hERG cells with a JAK1 inhibitor prevented IL-6 from decreasing *I_K__r_* [58]. Increased expression of the JAK2 gene has been found in obese guinea pig models fed with a PA-containing HFD [33]. Injection of a JAK2 agonist, coumermycin, in the same models led to an enhanced risk of VA [33]. In addition to JAKs, phosphorylation and nuclear translocation of their downstream proteins, called STATs, may also be involved in cardiometabolic arrhythmogenic changes, as evidenced by the enhanced phosphorylation and nuclear translocation of STAT4 in these animal models, which has been found to be strongly associated with the enhanced VA/SCD frequency observed in them [33]. Therefore, understanding the pathophysiology of IL-6 is imperative for targeting IL-6 pathways for therapeutic means.

FFAs exert a proinflammatory response by promoting IL-6 expression and the initiation of downstream IL-6 signaling [181]. Two crucial mechanisms by which FFAs initiate this signaling cascade are through interrelated pathways including Toll-like receptors (TLRs) and the NACHT, leucine-rich repeat (LRR)-, and pyrin domain (PYD)-containing protein 3 (NLRP3) inflammasome; both of these are part of the pattern recognition class of proteins belonging to the first line of defense in the body, which is known as innate immunity [181,182]. TLRs are transmembrane pattern recognition receptors that are able to respond and bind to pathogen-associated molecular patterns (PAMPs), and they are known to instigate an inflammatory state in cardiomyocytes by activating nuclear factor kappa B (NF-κB) [183]. While cardiomyocytes express most TLRs, TLR2 and TLR4 are commonly influenced by FFAs [181,183]. Qian et al. identified that inhibition of TLR2 by C29 (a TLR2 inhibitor) or TLR2-knock-out high-fat-diet-fed mice successfully reversed the elevated creatine kinase MB (CK-MB) and cytokine (IL-6, IL-1β, and TNF-α) levels, and it attenuated cardiac hypertrophy and myocardial fibrosis, confirming the role of TLR2 in obesity-mediated cardiomyopathy [184]. In addition, immunoblot analysis revealed that the decline in TLR2 expression did indeed result in NF-κB inhibition [184]. Further, immunoprecipitation assays showed that lipotoxicity augments TLR2 myeloid differentiation primary response 88 (MYD88) complex levels in a TLR4-independent manner rather than TLR2 alone [184]. The hypertrophic changes induced by TLR2 through NF-κB and IL-1β may be due to adaptive responses to pressure overload, as smaller cardiomyocytes, lower heart-weight-to-body-weight ratios, and lower fibrosis were observed in Tlr2−/− mice in a study by Higashikuni et al. [185]. Therefore, it can be concluded that TLR2 may be a potential therapeutic target in diastolic HF. Similarly, inhibition of TLR4 also mitigates metabolic-associated cardiomyopathy [186,187,188,189,190]. TLR4 and co-receptor myeloid differentiation protein 2 (MD2) mostly share a downstream pathway similar to that of TLR2, as they signal through the TLR/MYD88/NF-κB pathway [191]. TLR4 can additionally be activated independently from MYD88, which also leads to NF-κB activation [191].

The activation of NF-κB by pattern recognition receptors such as TLRs can lead to the “priming” of NLRP3. NLRP3 is an inflammasome modulating the production of active IL-1β and IL-18. The activation of NLRP3 comprises two distinct steps, called priming and triggering, which are responsible for the transcription and reorganization of the NLRP3 components, respectively [182]. Priming of the NLRP3 components (NLRP3, pro-IL-1β, and pro-IL-18) leads to an increase in caspase-1, which, in turn, positively stimulates IL-1β [182]. Elevated NLRP3 levels are observed in obesity-related vascular dysfunction [192]. Moreover, in a study by Ralston et al., it was demonstrated that extracellular ATP accumulation activates NLRP3 through P2X purinoceptor 7 (P2X7) [193], which is a protein known for inducing cellular K depletion and K efflux [194]. In addition to K, NLRP3 also holds an intricate relationship with Ca, as revealed by cytosolic Ca increases either through P2X7/K exchange or from the SR [182]. Furthermore, tirzepatide has demonstrated cardioprotective effects against sepsis-induced cardiomyopathy, evidenced by reduced APD and QTc, through targeting the TLR4/NF-kB/NLRP3 pathway [195]. These findings indicate that a deeper exploration of the relationship between IL-6, K, and Ca ionic properties and NLRP3 is warranted to gain a full understanding of the role of IL6 in HF.

### 4.2. Cardiac Bridging Integrator 1 Signaling in Heart Failure

Bridging integrator 1 (BIN1) is a BAR-domain-containing protein that is widely expressed in various tissues throughout the body [196]. Alternative splicing of the BIN1 gene results in tissue-specific BIN1 proteins [196]. The most frequently observed isoform in the heart is BIN1 + 13, which is responsible for cell proliferation [197]. However, the BIN1 + 13 + 17 isoform, which is formed by the combination of the cardiac alternatively spliced +13 and the ubiquitously alternatively spliced +17, is the cardiac isoform located on the T-tubules [198], and it mediates their densely folded morphology [198].

BIN1 + 13 + 17 is also known as cardiac bridging integrator 1 (cBIN1) and is a membrane scaffolding protein that creates micromembrane domains in cardiomyocytes; it is one of the critical structural proteins constituting cardiac dyads [199]. cBIN1 mediates T-tubule functions and cardiac Ca machinery by forming microfolds in the T-tubules [198,200], aiding in the clustering and forward-trafficking of the L-type calcium channel (LTCC) or Cav1.2 to T-tubules [201,202], recruiting RyRs to dyads [203], and eventually leading to RyR–Cav1.2 coupling at the dyads [196,203].

Due to its reported low expression in HF and re-tubulization ability, it has been postulated that cBIN1 has the potential to be used as a gene therapy agent for HFpEF [146,202,204,205,206]. cBIN1 gene therapy in diabetic mice with HFpEF has shown promising results by reversing T-tubule disarrangements and lowering glucose levels [29]. Chronic exposure to sympathetic stress by isoproterenol (beta agonist medication) induces concentric hypertrophy and disrupted Ca handling [204]. Exogenous cBIN1 replacement in mice undergoing 4 weeks of isoproterenol administration reversed hypertrophy in a study by Liu et al. [204]. The same study also indicated that cBIN1 gene therapy further attenuates the effects of pressure overload and mitigates diastolic dysfunction [204]. Loss of cBIN1 is also linked to arrhythmogenic alterations in failing cardiomyocytes [198]. Administration of augmented ventricular pacing to mice carrying heterozygous and homozygous deletions of BIN1 showed a higher prevalence of VF and VT arrhythmias compared with the control wild-type group, with a more severe VT being attributed to mice bearing the homozygous deletion of BIN1 [198]. Agreeing with these preclinical findings are the results of a clinical study by Hong et al. [205]. Utilizing an enzyme-linked immunosorbent assay (ELISA), Hong et al. found that a marked decrease (<30) in plasma BIN1 levels in patients with arrhythmogenic right ventricular cardiomyopathy can predict arrhythmia incidence with 82% and 79% sensitivity and accuracy, respectively [205].

As cBIN1 is a major factor influencing cardiac T-tubule function, disruptions in T-tubule folding are the primary reasons behind the cardiomyopathic effects of cBIN1 loss. While reduced cBIN1 may not change the expression level of Cav1.2, it leads to dysregulated trafficking of the channels to T-tubules [204]. A reduction in cBIN1 levels does not result in smaller T-tubules, but rather, causes diminished T-tubule folding and invaginations, creating an enlarged round lumen [198,204]. The diminished T-tubule folds disrupt the “fuzzy space” created by cBIN1 [198]. Fuzzy spaces are diffusion barriers that slow the ion diffusion taking place in the T-tubules, thereby stabilizing myocytes during ionic alterations [198]. Disruption of the diffusion barriers prompts heightened sensitivity to sympathetic stimulations and susceptibility to lethal arrhythmias [198]. In contrast, one study by Frisk et al. assessed ventricular tissues of patients undergoing coronary artery bypass graft surgery, and they found that although T-tubules were dilated and increased, BIN1 gene expression was not changed in HFpEF patients [110]. However, since BIN1 is alternatively spliced, a precise PCR isoform identification with real-time PCR confirmation is crucial for investigating the cBIN1 patterns in HFpEF [198,207].

Transmission electron microscopy findings by Xu et al. have revealed the presence of cBIN1-containing micro-particles (MPs) in mouse plasma [208]. Further analysis by Xu et al. indicated that these cBIN1-containing MPs originate from the T-tubule microfolds of ventricular cardiomyocytes [208]. Since MPs, like other vesicles, are subject to destruction when faced with adverse changes in osmolarity, Xu et al. induced hypotonic shock through the serial dilution of human plasma samples from heart failure patients, which led to a significant increase in the plasma concentrations of cBIN1 [208]. Lastly, the same study elucidated a strong and negative correlation between plasma cBIN1 levels and heart failure, evidenced by the reduced concentrations in HF patients compared with those in patients with normal heart conditions [208]. Given the availability of cBIN1 in plasma, it may serve as a potent biomarker in HFpEF patients. To this end, a normalized inverse index of the value of plasma cBIN1 levels called the cBIN1 score has been formulated [209]. The cBIN1 score is shown to be higher in HFpEF patients and is a promising diagnostic and prognostic biomarker for HFpEF [209].

### 4.3. Leukotriene B4 Signaling in Heart Failure

Leukotriene B4 (LTB4), a member of the leukotriene family, originates from the oxidation of arachidonic acid by 5-lipoxygenase (5-LO) [210]. LTB4 is a proinflammatory lipid mediator and leukocyte chemoattractant that is known for its significant role in insulin resistance and metabolic inflammation. Elevated levels thereof are extensively found in obese and diabetic preclinical models [151,152]. LTB4 signaling plays a key role in cardiovascular diseases, and there is increasing evidence in the extant literature that LTB4 signaling serves as a contributing factor and a potential biomarker in a wide range of cardiovascular diseases, including atherosclerosis, MI, and acute coronary syndrome [211,212,213,214,215,216]. Intriguingly, a recent study by Corbin et al. demonstrated an increased propensity to spontaneous arrhythmias along with a poor response to isoproterenol challenge, which included severe electrical conduction abnormalities resulting in heart block and asystole in LTB4-challenged guinea pigs [152]. In vitro administration of LTB4 to guinea pig ventricular myocytes further demonstrated a significant decline in *I_Kr_* current and a reduction in hERG1a surface expression levels, suggesting that LTB4 might be a potent modulator of cardiac electrical activity, making this lipid mediator a possible therapeutic target in metabolism-induced cardiac arrhythmias [152].

Interestingly, the inhibition of the LTB4 receptor (LTBR) instigates glucose tolerance, anti-inflammatory macrophage polarization, and cardioprotective effects against arrhythmogenic QT prolongations [151,152,217]. The cardioprotective benefits of LTB4 inhibition are further confirmed in a recent study on mice exposed to doxorubicin, which is a cardiotoxic chemotherapeutic agent with established alterations in Ca handling as a consequence [218]. Pre-treatment with empagliflozin in these mouse models increased the EF and reduced LTB4 production by arachidonic acid [219]. Thus, investigating other LTB4 inhibitors may provide promising targets for reversing cardiac remodeling. Moreover, a pronounced increase in LTB4 is evident in adipose tissue [217]. It is well documented that elevated adipose tissue levels are accountable for ventricular remodeling in HFpEF [16]. In particular, changes in EAT, due to its proximity to the ventricles and positive correlation with BMI values, greatly influence AP disturbances, fibrosis, and the overall outcome in HFpEF [220]. Furthermore, it is known that EAT partly exerts its effect by releasing proinflammatory cytokines such as IL-6, IL-1β, and TNFα [16], thus emphasizing the importance of investigating the LTB4 pathway and its mechanisms in metabolic inflammation.

The LTB4 pathway is initiated by LTB4 binding to its G-protein-coupled receptors, BLT1 and BLT2 [221]. Despite the high BLT2 gene expression observed in patients with atherosclerosis in a study by Sanchez-Galan et al. [222], BLT2 is generally more correlated with non-cardiovascular inflammation and holds a lower affinity for LTB4 than BLT1 [221,223]. On the other hand, the LTB4/BLT1 axis is directly involved in cardiac lipotoxicity and inflammation [151]. Flow cytometry analysis in high-fat-diet-fed mice showed an increased surface expression of BLT1, specifically on monocytes [217]. The same study revealed that BLT1-deficient mice demonstrated fewer M1 macrophages and lower IL-6 levels [217]. These findings indicate that LTB4 may serve as a chemoattractant for monocytes in a state of obesity. Additionally, studies investigating the role of the LTB4/BLT1 axis in atherosclerosis have demonstrated that LTB4 can also stimulate the production of monocyte chemoattractant protein-1 (MCP-1/CCL2) [211,224]. It is known that in obesity, monocytes, as part of the innate immune system, are recruited to adipocytes from the bloodstream and are subsequently polarized into M1 macrophages, which initiates a cascade leading to proinflammatory cytokine secretion and eventually insulin resistance [217,225,226]. Therefore, it can be concluded that monocyte trafficking, a crucial step in obesity-mediated lipotoxicity, is partly dependent on LTB4 [225]. Intriguingly, the inhibition of BLT1 in obese mouse models resulted in lower levels of M1 macrophages and proinflammatory cytokines, including IL-6 [151].

Another path by which LTB4 instigates the release of inflammatory cytokines is by enhancing the activity of TLRs [227,228]. Although it was found by Gaudreault et al. that LTB4 does not have a direct effect on the expression of TLRs [227], LTB4 influences the TLR/MyD88/NF-κB pathway by either increasing the expression of MyD88 through the positive regulation of miR-155 [228] or the phosphorylation of TGF-β-activated kinase 1 (TAK1), a downstream protein of TLR/MyD88, promoting cytokine production through the overexpression of NF-κB [151,227]. Moreover, TAK1 phosphorylation by LTB4 further contributes to cytokine infiltration through the mitogen-activated protein kinase (MAPK) pathway [151,227].

The notable correlations of LTB4 with insulin resistance and lipotoxicity suggest a role as a biomarker and a therapeutic role for LTB4 in metabolic HFpEF. Furthermore, higher LTB4 levels have been found in diabetic patients with cardiovascular autonomic neuropathy (CAN) [229]. CAN constitutes a state of dysfunctional nerve fibers serving the cardiovascular system [230]. CAN is a severe and common complication of diabetes that is strongly associated with long QT intervals, cardiac arrhythmias, and SCD in diabetic patients [230]. It is now widely believed that CAN is more a consequence of the chronic inflammation associated with metabolic syndrome rather than merely a complication of a hyperglycemic state [231]. In addition to inflammatory cytokines, factors indicating endothelial dysfunction, such as eNOS, NO, and NO bioavailability, are also linked with CAN incidence and outcomes in diabetic patients [232].

The elevated levels of LTB4 in patients with CAN and the high incidence of cardiac arrhythmias in these patients suggest that LTB4 inhibition may be a possible treatment for ventricular arrhythmias in these patients and, consequently, for diabetic patients developing HFpEF. Administration of zileuton, an LTB4 and cysteinyl leukotrienes inhibitor, has successfully shortened the duration of VAs in rats undergoing ischemia–reperfusion injury [233]. Conversely, montelukast, a cysteinyl leukotriene inhibitor, did not have such an effect [233]. Therefore, LTB4 stands as a potent target for VA treatment. Nevertheless, extensive investigation is needed to assess how LTB4 modulates the expression of cardiac ion channels and their resulting ion currents. This information is crucial to attaining a comprehensive evidence-based approach to utilizing LTB4 inhibitors for the treatment of VAs in metabolic HFpEF [234].

## 5. Conclusions and Future Directions

In conclusion, since the prevalence of HFpEF is increasing and the development of an effective treatment approach remains elusive, particular attention to this disease is warranted. Sudden cardiac death through VA poses a significant threat to HFpEF patients. An obesity-linked HFpEF phenotype is particularly common, with obesity not only being a comorbidity but also a significant risk factor for HFpEF; consequently, this prompts an examination of how obesity and metabolic inflammation may exacerbate the underlying pathophysiology of VA. Given the evident role of metabolic inflammation in HFpEF, exploring ion channel modulations in this context may provide novel insights into potential treatment approaches.

This study focuses on how cardiac ion channels and currents are influenced during lipotoxicity, inflammation, and HFpEF. It is postulated that identifying the specific currents and channels that are dysregulated in HFpEF paves the way for therapeutic advances in this field. This hypothesis is further supported by the inhibition of late *I_Na_* current by empagliflozin, AIP, and vericiguat, as well as the inhibition of NCX by ranolazine, in HFpEF [19,21,85]. The data are, however, still limited in this regard. It would be beneficial to investigate the utilization of these medications in animal models that more accurately mimic the expression of ion channels in humans. As we have discussed, mice differ from humans in most K currents, including *I_K__r_* [138]. This proposition is highlighted by pharmaceutical guidelines deeming hERG cardiotoxicity screening to be essential for new drugs [235].

Moreover, it is crucial to investigate novel therapeutic targets in HFpEF, leading to our discussion of IL-6, cBIN1, and LTB4, among which the LTB4 pathway remains underappreciated and deserves further investigation. LTB4 is involved in insulin resistance, a phenomenon that is crucial in the formation of diabetes and may also drive arrhythmogenic changes in diabetic patients and eventually lead to HF. Diabetes and obesity compose an inflammatory state leading to heightened secretion of cytokines such as IL-6 and, eventually, the formation of HFpEF. Thus, it could be intriguing to know the following: (1) how LTB4 and IL-6 trans-signaling would affect cardiomyocytes and cardiac channels; (2) if the administration of an LTB4 inhibitor would reverse cardiac remodeling in obese and diabetic patients; (3) the differential expression of LTB4 and IL-6 in various adipose tissues and macrophages; (4) whether LTB4 could potentially influence myocardial fibrosis and instigate reentry circuits and fatal arrhythmias. Furthermore, it is recommended that future studies utilize multi-omics approaches to uncover significant and crucial pathways behind HFpEF-associated VAs. Multi-omics techniques gather data from various molecular pathways by integrating a wide range of biological molecules (genomics, transcriptomics, proteomics, and metabolomics) and facilitate the identification of novel cellular pathways and drug discovery processes [236].

To advance our comprehension of these molecular mechanisms, there is an urgent need for preclinical models that can bridge the existing gaps in the extant literature. If successful, these studies have the potential to make HFpEF more manageable for both clinicians and patients dealing with this condition.

## Figures and Tables

**Table 1:** Altered functional expression of ion channels in HFpEF and/or obese animal models.

Current	Gene	mRNA	Protein	Current Density	Animal Model	HFpEF/Obese	Cardiac Tissue	QT_C_	Drug Tx (Effect)	Ref.
late *I_Na_*	*SCN5A*	NR	NR	↑	Mouse (C57BL/6J)	+/+	Ventricle	NR	Pre-incubation with empagliflozin (↓ current density)	[19]
		NR	NR	↔	Rat (SD)	−/+	Ventricle	↔ (↑QRS)	NR	[20]
		NR	NR	↑ (male ˃ female)	Mouse (db/db + Aldo)	+/+	Ventricle	NR	Empagliflozin (↓ APD prolongation, male + female) AIP (↓ APD prolongation, male + female) Vericiguat (↓ APD prolongation, female only)	[21]
Peak *I_Na_*	*SCN5A*	NR	NR	↔	Rat (SD)	−/+	Ventricle	↔ (↑QRS)	NR	[20]
		↔	NR	↑ *	Rat (WR)	−/+	Ventricle	NR	NR	[22]
*I*_*Ca*,*L*_	*CACNA1c*	↔	↑	↑	Rat (Dahl/SS)	+/−	Ventricle	NR	NR	[23]
		↑	NR	↑ *	Rat (WR)	−/+	Ventricle	NR	NR	[22]
		NR	↔	↑	Rat (WR)	+/−	Ventricle		NR	[24]
		NR	NR	↑	Rat (HHR)	+/−	Ventricle	↔ (↑QRS)	NR	[25]
		NR	↔	NR	Rat (WR)	−/+	Ventricle	NR	NR	[26]
		↓	NR	NR	Rat (WR, 15 weeks)	−/+	Ventricle	NR	NR	[27]
		↑	NR	NR	Rat (WR, 30 weeks)	−/+	Ventricle	NR	NR	[27]
		↔	NR	NR	Rat (WR, 45 weeks)	−/+	Ventricle	NR	NR	[27]
		NR	↔	NR	Rat (WR)	−/+	Ventricle	NR	NR	[28]
		NR	↔	NR	Mouse (db/db)	+/−	Ventricle	NR	cBIN1	[29]
		↔	NR	↑	Rat (Dahl/SS)	+/−	Ventricle	↑	NR	[14]
		NR	NR	↓ (male only)	Mouse (db/db+Aldo)	+/+	Ventricle	NR	Empagliflozin (↓ APD prolongation, male + female) AIP (↓ APD prolongation, male + female) Vericiguat (↓ APD prolongation, female only)	[21]
		↔	NR	NR	Rat (WR)	−/+	Ventricle	NR	NR	[30]
*I_to_*	*KCND2/3*, *KCNA4*	NR	NR	↔	Rat (WR)	+/−	Ventricle	NR	NR	[31]
		↔	NR	↑ *	Rat (WR)	−/+	Ventricle	NR	NR	[22]
		NR	NR	↓ (male + female)	Mouse (db/db + Aldo)	+/+	Ventricle	NR	Empagliflozin (↓ APD prolongation, male + female) AIP (↓ APD prolongation, male + female) Vericiguat (↓ APD prolongation, female only)	[21]
		NR	NR	↑	Mouse (C57BL6)	−/+	Ventricle	NR	NR	[32]
	*KCND2*	↔	↔	↓	Rat (Dahl/SS)	+/−	Ventricle	↑	NR	[14]
	*KCND3*	↓	↓	↓	Rat (Dahl/SS)	+/−	Ventricle	↑	NR	[14]
	*KCNA4*	↔	↔	↓	Rat (Dahl/SS)	+/−	Ventricle	↑	NR	[14]
*I_Kr_*	*hERG*	↔	↔	↓	Rat (Dahl/SS)	+/−	Ventricle	↑	NR	[14]
		↓	NR	NR	Rat (WR)	−/+	Ventricle	NR	NR	[22]
		NR	↓	↓	Guinea pig (HFD)	−/+	Ventricle	↑	NR	[33]
*I_Ks_*	*KCNQ1*, *KCNE1*	↓	NR	↓	Guinea pig (HFD)	−/+	Ventricle	↑	NR	[33]
*I_K_*_1_	*KCNJ2/12/14/4*	NR	NR	↓	Rat (WR)	+/−	Ventricle	NR	NR	[31]
		↔	↔	NR	Rat (Dahl/SS)	+/−	Ventricle	↑	NR	[14]
		↑	NR	↑ *	Rat (WR)	−/+	Ventricle	NR	NR	[22]
		NR	NR	↓ (male + female)	Mouse (db/db+Aldo)	+/+	Ventricle	NR	Empagliflozin (↓ APD prolongation, male + female) AIP (↓ APD prolongation, male + female) Vericiguat (↓ APD prolongation, female only)	[21]

↑, increased; ↓, decreased; ↔, no change; NR, not reported; Tx, treatment; SD, Sprague Dawley; WR, Wistar rats; Dahl/SS, Dahl salt sensitive; db/db, genetically diabetic mouse; Aldo, aldosterone; HFD, high-fat diet; QTc, QT interval corrected for heart rate; AIP, autocamtide-2-related inhibitory peptide; APD, action potential duration. Note that any changes that were not found to be statistically significant are displayed as “no change”. * Computer simulations were used for prediction.

**Table 2:** Altered functional expression of cardiac Ca-handling proteins in HFpEF and/or obese animal models.

Ca Handling Protein	mRNA	Protein	Animal Model	HFpEF/Obese	Cardiac Tissue	Drug Tx (Effect)	Ref.
SERCA2a	↑	NR	Rat (WR)	−/+	Ventricle	NR	[22]
	↓	↓	Rat (Dahl/SS)	+/−	Ventricle	Intraperitoneal Ranolazine (↑ expression)	[85]
	NR	↔	Rat (WR)	+/−	Ventricle	NR	[31]
	NR	↓	Rat (WR)	+/−	Ventricle	NR	[24]
	NR	↓	Rat (HHR)	+/−	Ventricle	NR	[25]
	NR	↔	Rat (WR)	−/+	Ventricle	NR	[26]
	↓	NR	Rat (WR, 15 weeks)	−/+	Ventricle	NR	[27]
	↑	NR	Rat (WR, 30 weeks)	−/+	Ventricle	NR	[27]
	↓	NR	Rat (WR, 45 weeks)	−/+	Ventricle	NR	[27]
	NR	↔	Rat (WR)	−/+	Ventricle	NR	[28]
	↑	NR	Rat (WR)	−/+	Ventricle	NR	[30]
	NR	↔	Rat (ZSF1)	+/+	Ventricle	NR	[108]
	NR	↔	Mouse (C57BL/6J)	* +/+	NR	NR	[109]
	NR	↓	Mouse (db/db)	+/−	Ventricle	cBIN1 (↑ expression)	[29]
	NR	↓	Rat (Dahl/SS)	+/−	Ventricle	NR	[110]
	NR	↓	Rat (ZSF1)	+/+	Ventricle	NR	[110]
RyR2	↑	NR	Rat (WR)	−/+	Ventricle	NR	[22]
	NR	↔	Rat (WR)	+/−	Ventricle	NR	[31]
	NR	NR	Rat (SD)	+/−	Ventricle	Dantrolene (↑ RyR2 inhibition)	[111]
	↔	NR	Rat (WR, 15 weeks)	−/+	Ventricle	NR	[27]
	↑	NR	Rat (WR, 30 weeks)	−/+	Ventricle	NR	[27]
	↔	NR	Rat (WR, 45 weeks)	−/+	Ventricle	NR	[27]
	↑	NR	Rat (WR)	−/+	Ventricle	NR	[30]
	NR	↔	Mouse (db/db)	+/−	Ventricle	cBIN1	[29]
NCX	↑	NR	Rat (WR)	−/+	Ventricle	NR	[22]
	↑	↔	Rat (Dahl/SS)	+/−	Ventricle	Intraperitoneal Ranolazine (↓ expression)	[85]
	NR	↔	Rat (WR)	+/−	Ventricle	NR	[31]
	NR	↔	Rat (WR)	+/−	Ventricle	NR	[24]
	NR	↔	Rat (HHR)	+/−	Ventricle	NR	[25]
	↓		Rat (WR, 15 weeks)	−/+	Ventricle	NR	[27]
	↑	NR	Rat (WR, 30 weeks)	−/+	Ventricle	NR	[27]
	↓	NR	Rat (WR, 45 weeks)	−/+	Ventricle	NR	[27]
	↔	NR	Rat (WR)	−/+	Ventricle	NR	[30]
	NR	↔	Rat (Dahl/SS)	+/−	Ventricle	NR	[110]
	NR	↓	Rat (ZSF1)	+/+	Ventricle	NR	[110]
PLB	NR	↔	Rat (WR)	+/−	Ventricle	NR	[31]
	NR	↔	Rat (WR)	+/−	Ventricle	NR	[24]
	NR	↓	Rat (HHR)	+/−	Ventricle	NR	[25]
	NR	↔	Rat (WR)	−/+	Ventricle	NR	[26]
	↓	NR	Rat (WR, 15 weeks)	−/+	Ventricle	NR	[27]
	↑	NR	Rat (WR, 30 weeks)	−/+	Ventricle	NR	[27]
	↔	NR	Rat (WR, 45 weeks)	−/+	Ventricle	NR	[27]
	NR	↓	Rat (WR)	−/+	Ventricle	NR	[28]
	↑	NR	Rat (WR)	−/+	Ventricle	NR	[30]
	NR	↑	Rat (ZSF1)	+/+	Ventricle	NR	[108]
	NR	↔	Rat (Dahl/SS)	+/−	Ventricle	NR	[110]
	NR	↔	Rat (ZSF1)	+/+	Ventricle	NR	[110]

↑, increased; ↓, decreased; ↔, no change; NR, not reported; Tx, treatment; WR, Wistar rats; Dahl/SS, Dahl salt-sensitive; ZSF1, Zucker fatty; db/db, a genetically diabetic mouse; SD, Sprague Dawley; HHR, hypertrophic heart rat; QTc, QT interval corrected for heart rate; cBIN1, cardiac bridging integrator 1. Note that any changes that were not found to be statistically significant are displayed as “no change”. * Diastolic dysfunction with no systolic dysfunction.

## Data Availability

Data sharing is not applicable to this article as no datasets were generated or analyzed during the current study. All of the relevant data are included within the paper itself.

## Nonstandard Abbreviations and Acronyms

HFpEF	Heart failure with preserved ejection fraction
HFrEF	Heart failure with reduced ejection fraction
SCD	Sudden cardiac death
EAT	Epicardial adipose tissue
*I_Na_*	Sodium current
*I*_*Ca*,*L*_	L-type calcium current
*I_to_*	Transient outward potassium current
*I_Kr_*	Rapid delayed rectifier potassium current
*I_Ks_*	Slow delayed rectifier potassium current
*I_K1_*	Inward rectifier potassium current
QTc	Corrected QT interval
NCX	Sodium–calcium exchanger
EAD	Early afterdepolarization
DAD	Delayed afterdepolarization
SGLT2	Sodium–glucose cotransporter-2
CamK	Calcium–calmodulin kinase
RyR	Ryanodine receptor
eNOS	Endothelial nitric oxide synthesis
NO	Nitric oxide
TNF	Tumor necrosis factor
TGF-β	Transforming growth factor-β
SERCA	Sarcoplasmic reticulum Ca^2+^-ATPase
PLB	Phospholamban
hERG	Human ether-a-go-go-related gene
VT	Ventricular tachycardia
VF	Ventricular fibrillation
IL-6R	Interleukin-6 receptor
gp130	Glycoprotein 130
JAK/STAT	Janus kinase/signal transducers and activators of transcription
TLR	Toll-like receptor
NLRP3	Leucine-rich repeat (LRR) and pyrin domain (PYD)-containing protein 3
NF-κB	Nuclear factor kappa B
MyD88	Myeloid differentiation primary response 88
FFA	Free fatty acid
LTB4	Leukotriene B4
BLT1/2	Leukotriene B4 receptor 1/2
cBIN1	Cardiac bridging integrator 1
